# Supplementary material for: Sensitivity of Immunodiagnostic Tests in Localized Versus Disseminated Tuberculosis—A Systematic Review of Individual Patient Data
Source: Trop Med Infect Dis. 2025 Mar 7;10(3):70. doi: 10.3390/tropicalmed10030070 (PMC11946558; doi:10.3390/tropicalmed10030070)
Supplement: Supplementary file 1 [file tropicalmed-10-00070-s001.zip › tropicalmed-3465159-supplementary.pdf]

Supplementary Table S1: PRISMA-IPD Checklist of items to include when reporting a systematic review and meta-analysis of individual participant data (IPD)

PRISMA-IPD Section/topic	Item No	Checklist item	**Reported on page**
Title			
Title	1	Identify the report as a systematic review and meta-analysis of individual participant data.	Page 1
Abstract			
Structured summary	2	Provide a structured summary including the following, as applicable:	Page 1
		Background: State research question and main objectives, with information on participants, interventions, comparators and outcomes.	
		Methods: Report eligibility criteria; data sources including dates of last bibliographic search or elicitation, noting that IPDs were sought; methods of assessing risk of bias.	
		Results: Provide number and type of studies and participants identified and number (%) obtained, and summary effect estimates for main outcomes (benefits and harms) with confidence intervals and measures of statistical heterogeneity. Describe the direction and size of the summary effects in terms of those that are meaningful to those who would put findings into practice.	
		Discussion: State main strengths and limitations of the evidence, the general interpretation of the results and any important implications.	
		Other: Report the primary funding source, registration number and registry name for the systematic review and IPD meta-analysis.	
Introduction			
Rationale	3	Describe the rationale for the review in the context of what is already known.	Page 2, line 47-71
Objectives	4	Provide an explicit statement of the questions being addressed with references, as applicable, to participants, interventions, comparisons, outcomes and study design (PICOS). Include any hypotheses that relate to particular types of participant-level subgroups.	Page 2, line 73 -81
Methods			
Protocol and	5	Indicate if a protocol exists and where it can be accessed. If available, provide registration information including the registration number and registry name. Provide publication details, if applicable.	Page 3, line 91-96

registration			
Eligibility criteria	6	Specify inclusion and exclusion criteria, including those relating to participants, interventions, comparisons, outcomes, study design and characteristics (e.g., years when conducted, required minimum follow-up). Note whether these were applied at the study or individual level, i.e., whether eligible participants were included (and ineligible participants were excluded) from a study that included a wider population than specified by the review inclusion criteria. The rationale for criteria should be stated.	Page 3, line 89- page 4 line 145
Identify studies' information sources	7	Describe all methods of identifying published and unpublished studies including, as applicable, which bibliographic databases were searched with dates of coverage; details of any hand searching including of conference proceedings; use of study registers and agency or company databases; contact with the original research team and experts in the field; open adverts and surveys. Give the date of last search or elicitation.	Page 4, line 147 - 168.
Identify the study search	8	Present the full electronic search strategy for at least one database, including any limits used, such that it could be repeated.	Page 4, line 148-152
Study selection processes	9	State the process for determining which studies were eligible for inclusion.	Page 3, line 113 to page 4, line 145
Data collection processes	10	Describe how IPDs were requested, collected and managed, including any processes for querying and data with investigators. If IPDs were not sought from any eligible study, the reason for this should be stated (for each such study).<br>If applicable, describe how any studies for which IPDs were not available were dealt with. This should include whether, how and what aggregate data were sought or extracted from study reports and publications (such as extracting data independently in duplicates) and any processes for obtaining and confirming these data with investigators.	Page 4, line 155 to 163
Data items	11	Describe how the information and variables to be collected were chosen. List and define all study level and participant level data that were sought, including baseline and follow-up information. If applicable, describe methods of standardizing or translating variables within the IPD datasets to ensure common scales or measurements across studies.	Page 4, line 164-168
IPD integrity	A1	Describe what aspects of IPDs were subject to data checking (such as sequence generation, data consistency and completeness, baseline imbalance) and how this was done.	Not applicable
Risk of bias assessment in individual studies	12	Describe methods used to assess the risk of bias in the individual studies and whether this was applied separately for each outcome. If applicable, describe how findings of IPD checking were used to inform the assessment. Report if and how risk of bias assessment was used in any data synthesis.	Page 5, Line 207 to page 6, line 217

Specification of outcomes and effect measures	13	State all treatment comparisons of interest. State all outcomes addressed and define them in detail. State whether they were pre-specified for the review and, if applicable, whether they were primary/main or secondary/additional outcomes. Give the principal measures of effect (such as the risk ratio, hazard ratio, and difference in means) used for each outcome.	Page 5, line 170-177.
Synthesis methods	14	Describe the meta-analysis methods used to synthesize IPDs. Specify any statistical methods and models used. Issues should include (but are not restricted to) the following:<br>• Use of a one-stage or two-stage approach.<br>• How effect estimates were generated separately within each study and combined across studies (where applicable).<br>• Specification of one-stage models (where applicable) including how the clustering of patients within studies was accounted for.<br>• Use of fixed or random effects models and any other model assumptions, such as proportional hazards.<br>• How (summary) survival curves were generated (where applicable).<br>• Methods for quantifying statistical heterogeneity (such as I^2 and τ^2).<br>• How studies providing IPDs and not providing IPDs were analyzed together (where applicable).<br>• How missing data within the IPDs were dealt with (where applicable).	Page 5, line 180-197
Exploration of variations in effects	A2	If applicable, describe any methods used to explore variations in effects by study or participant level characteristics (such as estimations of interactions between effects and covariates). State all participant-level characteristics that were analyzed as potential effect modifiers, and whether these were pre-specified.	Page 5, line 199-205
Risk of bias across studies	15	Specify any assessment of risk of bias relating to the accumulated body of evidence, including any pertaining to not obtaining IPDs for particular studies, outcomes or other variables.	Page 5, line 206 to page 6, line 217
Additional analyses	16	Describe methods of any additional analyses, including sensitivity analyses. State which of these were pre-specified.	n/a
**Results**			
Study selection and IPD obtainment	17	Give numbers of studies screened, assessed for eligibility, and included in the systematic review with reasons for exclusions at each stage. Indicate the number of studies and participants for which IPDs were sought and for which IPDs were obtained. For those studies where IPDs were not available, give the numbers of studies and participants for which aggregate data were available. Report reasons for the non-availability of IPDs. Include a flow diagram.	See figure 1, page 7
Study characteristics	18	For each study, present information on key study and participant characteristics (such as descriptions of interventions, numbers of participants, demographic data, unavailable outcomes, funding sources, and if applicable, durations of follow-up). Provide (main) citations for each study. Where applicable, also report similar study characteristics for any studies not providing IPDs.	See page 8 and page 9

IPD integrity	A3	Report any important issues identified in checking IPDs or state that there were none.	None
Risk of bias within studies	19	Present data on risk of bias assessments. If applicable, describe whether data-checking led to the up-weighting or down-weighting of these assessments. Consider how any potential bias impacts the robustness of meta-analysis conclusions.	Page 10, line 356 to page 11, line 369.
Results of individual studies	20	For each comparison and for each main outcome (benefit or harm) for each individual study, report the number of eligible participants for which data were obtained and show simple summary data for each intervention group (including, where applicable, the number of events), effect estimate and confidence interval. These may be tabulated or included on a forest plot.	Results, paragraph 2-5, Table 3, Supplementary Table 2
Results of syntheses	21	Present summary effects for each meta-analysis undertaken, including confidence intervals and measures of statistical heterogeneity. State whether the analysis was pre-specified, and report the numbers of studies and participants and, where applicable, the number of events on which it was based.	See pages 8,9 and 10
		When exploring variations in effects due to patient or study characteristics, present summary interaction estimates for each characteristic examined, including confidence intervals and measures of statistical heterogeneity. State whether the analysis was pre-specified. State whether any interaction is consistent across trials.	
		Provide a description of the direction and size of effects in terms that are meaningful to those who would put these findings into practice.	
Risk of bias across studies	22	Present results of any assessment of risk of bias relating to the accumulated body of evidence, including any pertaining to the availability and representativeness of available studies, outcomes or other variables.	Page 10, line 356 to page 11, line 369.
Additional analyses	23	Give results of any additional analyses (e.g., sensitivity analyses). If applicable, this should also include any analyses that incorporate aggregate data for studies that do not have IPDs. If applicable, summarize the main meta-analysis results following the inclusion or exclusion of studies for which IPDs were not available.	n/a
Discussion			
Summary of evidence	24	Summarize the main findings, including the strength of evidence for each main outcome.	See page 11, line 372-375 and page 12, line 430 – 433.
Strengths and	25	Discuss any important strengths and limitations of the evidence including the benefits of access to IPDs and any limitations arising from IPDs that were not available.	Page 12, line 415-429.

limitations			
Conclusions	26	Provide a general interpretation of the findings in the context of other evidence.	page 12, line 430 – 433.
Implications	A4	Consider the relevance to key groups (such as policy makers, service providers and service users). Consider implications for future research.	Not applicable
Funding			
Funding	27	Describe sources of funding and other support (such as the supply of IPDs), and the role in the systematic review of those providing such support.	Page 13, line 442

A1 – A3 denote new items that are additional to standard PRISMA items. A4 has been created as a result of re-arranging the content of the standard PRISMA statement to suit the way that systematic review IPD meta-analyses are reported.

© Reproduced with the permission of the PRISMA IPD Group, which encourages sharing and reuse for non-commercial purposes.

Supplementary Table S2: STROBE check list: STROBE Statement—Checklist of items that should be included in reports of ***case–control studies.***

	Item No	Recommendation	Reported on Page Number/Line Number	Reported in Section/Paragraph
**Title and abstract**	1	(*a*) Indicate the study's design with a commonly used term in the title or the abstract.	Page 1, line 3 and 4.	
		(*b*) Provide in the abstract an informative and balanced summary of what was done and what was found.	Page 1, line 24-41.	
**Introduction**				
Background/rationale	2	Explain the scientific background and rationale for the investigation being reported.	Page 2, line 47-71.	
Objectives	3	State specific objectives, including any prespecified hypotheses.	Page 2, line 73 - 81.	
**Methods**				
Study design	4	Present key elements of study design early in the paper.	Page 3, line 84-88.	
Setting	5	Describe the setting, locations, and relevant dates, including periods of recruitment, exposure, follow-up, and data collection.	Not applicable.	
Participants	6	(*a*) Give the eligibility criteria, and the sources and methods of case ascertainment and control selection. Give the rationale for the choice of cases and controls.	Page 3, line 89-page 4 line 145.	
		(*b*) For matched studies, give matching criteria and the number of controls per case.	Not applicable.	
Variables	7	Clearly define all outcomes, exposures, predictors, potential confounders, and effect modifiers. Give diagnostic criteria, if applicable.	Page 3, line 114 to page 4, line 140; page 5, line 170 –line 177.	

Data sources/ measurement	8*	For each variable of interest, give sources of data and details of methods of assessment (measurement). Describe the comparability of assessment methods if there is more than one group.	Page 4, line 147 -168.
Bias	9	Describe any efforts to address potential sources of bias.	Page 5, line 207 to Page 6, line 217.
Study size	10	Explain how the study size was arrived at.	Page 6, line 219 -241.
Quantitative variables	11	Explain how quantitative variables were handled in the analyses. If applicable, describe which groupings were chosen and why.	Page 5, line 180 – line 205.
Statistical methods	12	(*a*) Describe all statistical methods, including those used to control for confounding.	Page 5, line 180-197.
		(*b*) Describe any methods used to examine subgroups and interactions.	Page 5, line 189-192 and line 200-205.
		(*c*) Explain how missing data were addressed	Page 6, line 213-217.
		(*d*) If applicable, explain how the matching of cases and controls was addressed.	Not applicable.
		(*e*) Describe any sensitivity analyses.	Not applicable.
**Results**			
Participants	13*	(a) Report numbers of individuals at each stage of study—e.g., numbers considered potentially eligible, examined for eligibility, confirmed as eligible, included in the study, that completed follow-up, and were analyzed.	Page 6, line 247-249. Page 7 (PRISMA flow diagram).
		(b) Give reasons for non-participation at each stage.	Page 7 (PRISMA flow diagram).
		(c) Consider the use of a flow diagram.	Page 7.

Descriptive data	14*	(a) Give characteristics of study participants (e.g., demographic, clinical, social) and information on exposures and potential confounders.	Page 8.
		(b) Indicate the number of participants with missing data for each variable of interest.	Page 10 in legend to Table 3; page 11, line 362-369.
Outcome data	15*	Report the numbers in each exposure category, or summary measures of exposure.	Page 8, Table 1; page 9, Table 2.
Main results	16	(*a*) Give unadjusted estimates and, if applicable, confounder-adjusted estimates and their precision (e.g., a 95% confidence interval). Make clear which confounders were adjusted for and why they were included.	Not applicable.
		(*b*) Report category boundaries when continuous variables were categorized.	Not applicable.
		(*c*) If relevant, consider translating estimates of relative risk into absolute risk for a meaningful time period.	Not applicable.
Other analyses	17	Report other analyses done—e.g., analyses of subgroups and interactions, and sensitivity analyses. -Page 8, Table 1; page 9 Table 2; page 10, Table 3; page 14, Supplementary Table 2.	
**Discussion**			
Key results	18	Summarize key results with references to study objectives. Page 11, line 372 to page 12, line 429.	
Limitations	19	Discuss limitations of the study, taking into account sources of potential bias or imprecision. Discuss both the direction and magnitude of any potential bias. Page 12, line 416 to 429.	
Interpretation	20	Give a cautious overall interpretation of results considering objectives, limitations, a multiplicity of analyses, results from similar studies, and other relevant evidence. Page 12, line 43-433.	
Generalizability	21	Discuss the generalizability (external validity) of the study results. Page 11, line 375-386.	
**Other information**			
Funding	22	Give the sources of funding and the roles of the funders for the present study and, if applicable, for the original study on which the present article is based. Not applicable.	

*Give information separately for cases and controls.

Note: An explanation and elaboration article discusses each checklist item and gives methodological background and published examples of transparent reporting. The STROBE checklist is best used in conjunction with this article (freely available on the Websites of PLoS Medicine at http://www.plosmedicine.org/, Annals of Internal Medicine at http://www.annals.org/, and Epidemiology at http://www.epidem.com/). Information on the STROBE Initiative is available at http://www.strobe-statement.org.

Supplementary Table S3: Composition of patients with immunosuppression in groups compared.

	Patients with lymph node tuberculosis	Patients with disseminated tuberculosis	P-value
**Patients from high-income countries (n[a])**	27	51	n.a.
Immune-mediated disease on anti-TNF treatment (n)	1	9	0.151
Immune-mediated disease on Anti-B-cell treatment (n)	0	1	1.000
Immune-mediated disease on immunosuppressive drugs (steroids or chemotherapy)(n)	1	2	1.000
Diseases associated with bone marrow failure (n)	1	2	1.000
Chronic renal failure on dialysis (n)	3	2	0.334
**Patients from less-than-high-income countries (n[a])**	22	38	n.a.
Immune-mediated disease on anti-TNF treatment (n)	0	0	1.000
Immune-mediated disease on Anti-B-cell treatment (n)	0	1	1.000
Immune-mediated disease on immunosuppressive drugs (steroids or chemotherapy)(n)	0	1	1.000
Diseases associated with bone marrow failure or a genetic condition causing reduced interferon gamma production (n)	2	0	0.132

Chronic renal failure on dialysis (n)	0	1	1.000

[a] This refers to the number of patients for whom information on co-morbidity was available.
